# Unveiling the role of PYGB in pancreatic cancer: a novel diagnostic biomarker and gene therapy target

**DOI:** 10.1007/s00432-024-05644-2

**Published:** 2024-03-14

**Authors:** Li-kun Ren, Ri-shang Lu, Xiao-bin Fei, Shao-jie Chen, Peng Liu, Chang-hao Zhu, Xing Wang, Yao-zhen Pan

**Affiliations:** 1 College of Clinical Medicine, Guizhou Medical University, Guiyang, 550000 Guizhou China; 2 Department of Hepatic-Biliary-Pancreatic Surgery, The Affiliated Hospital of Guizhou Medical University, Guiyang, 550000 China; 3 Department of Hepatic-Biliary-Pancreatic Surgery, The Affiliated Cancer Hospital of Guizhou Medical University, Guiyang, 550000 China

**Keywords:** Brain glycogen phosphorylase (PYGB), Pancreatic cancer, Proliferation, Metastasis, MAPK/ERK, Bioinformatics analysis

## Abstract

**Purpose:**

Pancreatic cancer (PC) is a highly malignant tumor that poses a severe threat to human health. Brain glycogen phosphorylase (PYGB) breaks down glycogen and provides an energy source for tumor cells. Although PYGB has been reported in several tumors, its role in PC remains unclear.

**Methods:**

We constructed a risk diagnostic model of PC-related genes by WGCNA and LASSO regression and found PYGB, an essential gene in PC. Then, we explored the pro-carcinogenic role of PYGB in PC by in vivo and in vitro experiments.

**Results:**

We found that PYGB, SCL2A1, and SLC16A3 had a significant effect on the diagnosis and prognosis of PC, but PYGB had the most significant effect on the prognosis. Pan-cancer analysis showed that PYGB was highly expressed in most of the tumors but had the highest correlation with PC. In TCGA and GEO databases, we found that PYGB was highly expressed in PC tissues and correlated with PC's prognostic and pathological features. Through in vivo and in vitro experiments, we found that high expression of PYGB promoted the proliferation, invasion, and metastasis of PC cells. Through enrichment analysis, we found that PYGB is associated with several key cell biological processes and signaling pathways. In experiments, we validated that the MAPK/ERK pathway is involved in the pro-tumorigenic mechanism of PYGB in PC.

**Conclusion:**

Our results suggest that PYGB promotes PC cell proliferation, invasion, and metastasis, leading to poor patient prognosis. PYGB gene may be a novel diagnostic biomarker and gene therapy target for PC.

**Supplementary Information:**

The online version contains supplementary material available at 10.1007/s00432-024-05644-2.

## Introduction

Pancreatic cancer(PC) is currently the third leading cause of cancer-related deaths worldwide; it is the malignant tumor whose mortality rate is closest to the incidence rate, with a 5-year survival rate of approximately 10% (Halbrook et al. 2023; Siegel et al. 2022; Sung et al. 2021), indicating that it seriously endangers human life and health. The early symptoms of PC are nonspecific, which makes the diagnosis difficult, and the majority of patients are already in advanced stages at the time of diagnosis. At this point, they have lost the chance to undergo radical surgery (Xia et al. 2022). Since it is rare for PC patients to be diagnosed early because the disease lacks specific clinical symptoms and early screening methods, targeted therapy is a crucial part of treating PC (da Paixão et al. 2022). Thus, it is imperative to investigate the biological characteristics and molecular pathways underlying PC.

Cancer cells consume glucose as essential nutrients to rapidly increase proliferation (Walker-Samuel et al. 2013). Glucose is transported into cancer cells, phosphorylated to glucose-6 phosphate (G6P), and then localized (Meng et al. 2023). G6P involves multiple metabolic pathways in tumors, including glycogen metabolism and glycolysis (Pavlova and Thompson 2016). In a normoxic setting, G6P produced via glycogen metabolism fuels the glycolytic pathway in neoplastic cells, elevating lactate production. This phenomenon is called aerobic glycolysis or the Warburg effect (DeBerardinis and Chandel 2020). Metabolic alteration contributes to the accelerated remodeling of the local tumor immunological microenvironment (Pavlova et al. 2022). Glycogen metabolism is, therefore, critically important to tumor growth (Hindson 2021). This soluble macromolecule constitutes the most common form of glucose storage in the cytoplasm and is mainly broken down by glycogen phosphorylase (PYG) (Roach et al. 2012). The glycogen branches are linked together through α-1,4 glycosidic linkages, which can be enzymatically hydrolyzed by phosphorylase glycogen (PYG), resulting in the production of glucose-1-phosphate, which is further converted to G6P and is involved in various metabolic pathways, such as aerobic glycolysis in tumors (Favaro and Harris 2012). There are three different isoforms of PYG in humans, namely brain glycogen phosphorylase (PYGB), muscle glycogen phosphorylase (PYGM), and liver glycogen phosphorylase (PYGL) (Chrysina 2010). It is possible to develop glycogen storage diseases, significant hepatomegaly, and hepatocellular carcinoma when several glycogenolytic enzymes, such as PYGL and PYGB, are lost or mutated (Chou et al. 2010; Kim et al. 2017; Mutel et al. 2011; Resaz et al. 2014; Wilson et al. 2019). PYGB is a glycogen phosphorylase primarily present in the brain and plays a crucial role in glycogen metabolism (Mathieu et al. 2016). Interestingly, PYGB is highly expressed in various cancers, such as ovarian cancer (Zhou et al. 2019), glioblastoma (Ferraro et al. 2022), prostate cancer (Wang et al. 2018), and non-small cell lung cancer (Xiao et al. 2020). Notably, the relevance of PYGB in PC remains unclear.

A signaling system known as mitogen-activated protein kinases (MAPKs) regulates cell proliferation, differentiation, and death (Guo et al. 2020). There are four distinct subfamilies of this pathway, namely, ERK, p38, JNK, and BMK1 (Rovida and Tusa 2022). Among these subfamilies, the MAPK/ERK pathway is the most well-established and well-studied (Guo et al. 2020). Approximately 75–90% of PCs have RAS gene mutations, and the abnormal activation of RAS further activates downstream RAF, which then sequentially activates MEK and ERK, constituting the MAPK cascade reaction (Liu et al. 2018). After activation of ERK, it can enter the cell nucleus and affect the expression of c-myc, FoxO3, bcl-2, Bax, and other genes through phosphorylated transcription factors in the cell nucleus, thus promoting the progression of PC (Bryant et al. 2019).

**Fig. 1:**
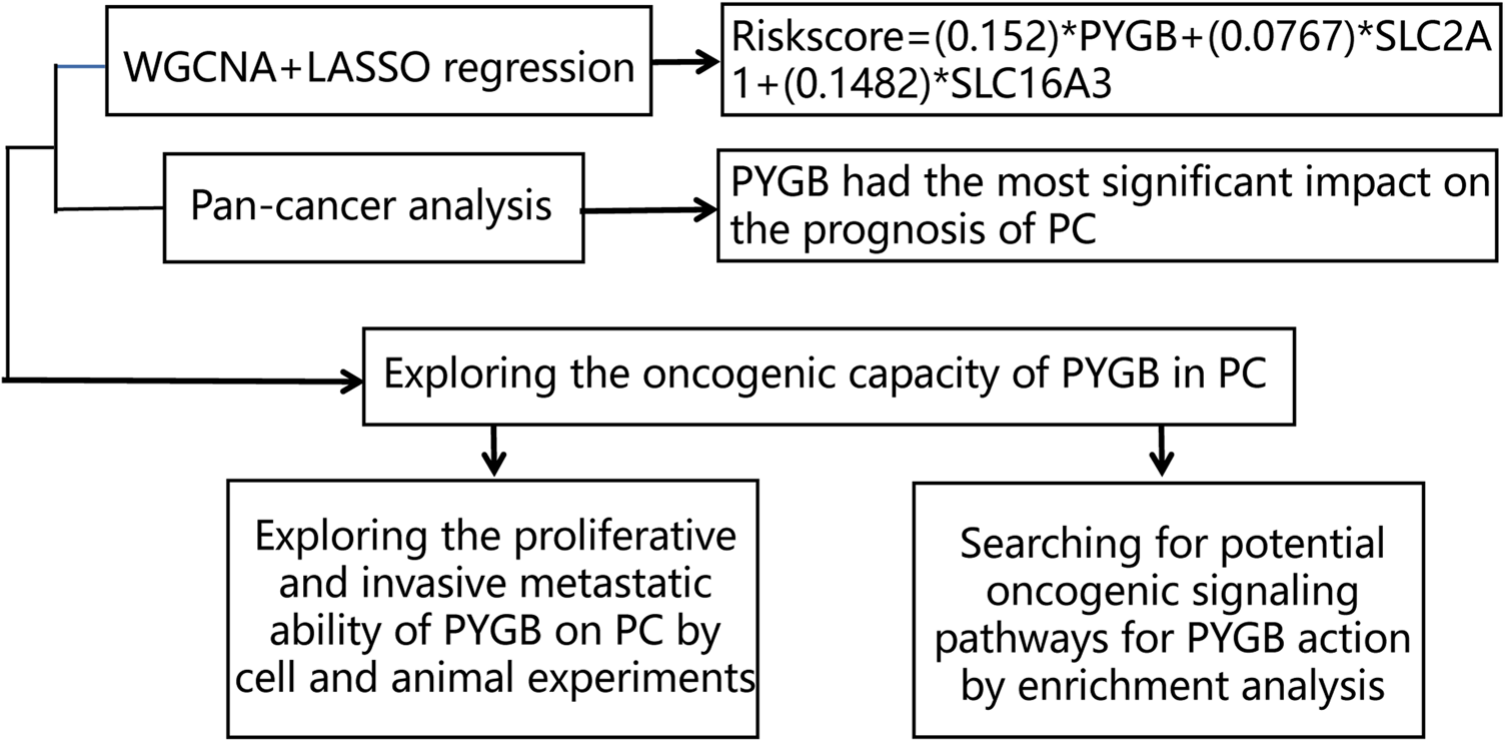
Flowchart of the study design. We identified an oncogenic factor, PYGB, in PC by WGCNA and LASSO regression models. PYGB was then found to be highly expressed in almost all tumors by pan-cancer analysis but had the most significant impact on the prognosis of PC. Then, we verified the effects of PYGB on PC’s proliferation, invasion, and metastasis through a series of cell and animal experiments and found the potential oncogenic mechanism of PYGB in PC through enrichment analysis

In this study, we screened essential genes for PC by WGCNA and constructed a prognostic risk model using LASSO regression. Combined with pan-cancer analysis, we found that PYGB had the most significant impact on PC. Moreover, we demonstrated that PYGB affects the MAPK/ERK signaling, promoting PC proliferation, invasion, and metastasis. PYGB may be a promising therapeutic target based on these studies. The study flowchart is shown in Fig. 1.

## Materials and methods

### Screening of PC-related genes using weighted gene co-expression network analysis (WGCNA)

We used the WGCNA method to generate weighted co-expression networks using the GSE16515 dataset and the GSE28735 dataset from the Gene Expression Omnibus database (GEO, http://www.ncbi.nlm.nih.gov/geo/) (Barrett et al. 2013). In this study, we used tumor versus non-tumor as the sample phenotype. We then selected appropriate soft-threshold power (*β*) values to generate scale-free networks (scale-free topology index of approximately 0.9). Then, similar genes were introduced into the same candidate module using the “dynamic tree-cutting” algorithm with a module size of 30. The Pearson correlation test performed correlation analysis between the module feature genes and the sample traits. We extracted the modules with the most significant correlations with tumors and further screened critical genes in the model. Finally, we screened 591 genes related to tumor energy metabolism in the GSEA database (http://www.gsea-msigdb.org/gsea/index.jsp) (Subramanian et al. 2005). We took the intersection of these genes with essential tumor-associated genes in the GSE16515 and GSE28735 datasets to discover essential genes for PC.

### Construction of PC‑related prognostic signature

First, we extracted the RNA-seq information of PC and patient prognosis information from The Cancer Genome Atlas (TCGA, https://portal.gdc.com) database (Tomczak et al. 2015). We screened the prognosis-related differential genes by univariate COX regression analysis on overall survival (OS). We then applied LASSO regression to construct a lambda-Min-based multigene signature containing the screened prognostic differential genes. The optimal value of lambda was determined by tenfold cross-validation. Univariate Cox regression and LASSO regression were performed in R (version 4.2.1) using the “survival” and “glmnet” packages. All samples were categorized into high and low-risk groups based on the optimal cutpoint value determined by the “surv_cutpoint” function in the R package “survminer”. The “surv_cutpoint” function in the R package “survminer” uses the maxstat (maximum selective rank statistic) statistic to determine the optimal cutpoint for continuous variables.

### Pan-cancer analysis of PYGB

First, we used the TIMER database (https://cistrome.shinyapps.io/timer/) (Li et al. 2017) to compare the expression of PYGB in human malignant tumors and related normal tissues. Subsequently, we acquired and arranged the RNA-seq data about the TCGA-ALL (pan-cancer) project from TCGA. The data were retrieved in the transcripts per million (TPM) format, along with the corresponding clinical information. For analysis and visualization, we employed the “ggplot2” package. Second, we used univariate Cox regression and constructed forest plots with the “forestplot” package to demonstrate the effects of PYGB on OS, progression-free interval (PFI), disease-specific survival (DSS), and disease-free survival (DFS) for each cancer type. Finally, we examined the differences in PYGB protein levels between tumors and normal tissues using the Human Protein Atlas (HPA, http://www.proteinatlas.org/) (Uhlen et al. 2017). The complete names and acronyms of various cancers are shown in Supplementary Table 1.

### The expression of PYGB in PC and its relationship with clinical prognosis

We obtained corresponding clinical and mRNA information for PYGB in 171 paraneoplastic samples and 179 PC samples from the GTEx and TCGA databases. We also obtained three microarray expression datasets: GSE62452, GSE16515, and GSE28735. PYGB expression in PC and its relationship with clinicopathological parameters were analyzed using the “ggplot2” software package. In addition, we carried out proportionate risk hypothesis testing and fitted survival regressions applying the “survival” package, and the outcomes were visualized using the “ggplot2” package and the “survminer” package. A proteomic analysis of normal pancreatic tissues and PC was likewise executed using UALCAN (https://ualcan.path.uab.edu/index.html) (Chandrashekar et al., 2022). Finally, we collected the genes in the corresponding pathways and analyzed them by the “GSVA” package, selecting the parameter method “ssgsea”, and finally analyzed the correlation between PYGB genes and pathway scores by Spearman correlation analysis.

### Functional enrichment analysis

After identifying 150 genes related to PYGB using the STRING (https://cn.string-db.org) database (Szklarczyk et al. 2023), these genes were analyzed by KEGG/GO enrichment using the “clusterProfiler” package. GSEA was then performed using the log2(fold change) values of all differentially expressed genes between the high and low PYGB expression groups, with the following reference gene set: c2.cp.all. v2022.1.Hs.symbols.gmt [All Canonical Pathways]. The enrichment analysis results were visualized using the “ggplot2” package.

### Human tissue collection

Tumor and surrounding normal tissues were collected from eight patients with PC at Guizhou Medical University Hospital. These patients did not receive preoperative chemotherapy, radiotherapy, biotherapy, or traditional Chinese medicine.

### Cell culture and transfection process

The PC cell lines AsPC-1, BxPC-3, MIA PaCa-2, PANC-1, SW1990, and HPDE6-C7 cells were purchased from the ATCC cell bank (USA). DMEM that contained antibiotics and 10% fetal bovine serum was used to culture PANC-1 cells and MIA PaCa-2 cells. Other cells were cultivated in a medium of RPMI 1640. All cells used for the experiments were cultured in a constant temperature incubator at 37 °C containing 95% air and 5% CO_2_. The siRNA sequences were as follows: PYGB siRNA-NC sequence: 5′-CCUAAGGUUAAGUCGCCCUCG-3′, PYGB siRNA-1# sequence: GGUCCUGUAUCCAAAUGAU, PYGB siRNA-2# sequence: 5′-CCCUGUACAAUCGAAUCAA-3′, PYGBsiRNA-3# sequence: 5′-CUGCUGAUGAAGCCAUCUAU-3′. Small interfering RNAs (siRNAs) were transfected into cells with Lipofectamine 3000 (Invitrogen, Carlsbad, CA, USA). The design and manufacture of lentiviral vectors, including the negative control, PYGB overexpression, and PYGB-encoding short hairpin RNA (shRNA), were carried out by GeneChem (China). All infection and transfection steps were followed rigorously.

### qRT‒PCR procedure

TRIzol reagent (Invitrogen, CA, USA) isolated RNA from PC cell lines and tissues. Complementary DNA (cDNA) was generated through reverse transcription, which was then employed in subsequent experimental procedures. Amplification of DNA was conducted with TB Green^®^ Premix Ex TaqTM (Takara, Japan). Afterward, the CFX96TM real-time system (Bio-Rad, California, USA) was used to measure the amplification of each gene. The primers utilized in the present investigation were PYGB, with the sense primer sequence of 5′-ACGCAGCAGCACTACTAC-3′ and the antisense primer sequence of 3′-TCGCAGGCATTCTGAAGG-5′. The α-tubulin gene sequence consists of a sense strand sequence of 5'-ACCAACCTGGTGCCCTATCC-3' and an antisense strand sequence of 5'-CAAGCATTGGTGATCT. α-Tubulin was used as an endogenous control. The results were determined using the 2^− ΔΔ*Ct*^ method.

### Western blotting

We first separated proteins from tissues or cells using RIPA lysis buffer (Merck Millipore, Waltham, MA, USA) and then assayed the corresponding protein concentrations employing BCA kits (Solarbio Co.). After electrophoresis of the samples, we transferred them to polyvinylidene difluoride (PVDF) membranes supplied by Merck Millipore. These membranes were then incubated for 2 h in a solution containing 5% skim milk. After adding the primary antibody solution to the samples, they were incubated overnight at 4°C. After a 2-h incubation with the secondary antibody, the membranes were washed three times with TBST. The bands were then observed. We used the imaging system from Bio-Rad Laboratories (Hercules, CA, USA). The following antibodies were used: anti-PYGB (1:1000, Proteintech, #12075), anti-MEK1/2 (1:5000, Proteintech, #11049), anti-ERK1/2 (1:2000, Proteintech, #11257), anti-P-ERK1/2 (1:1000, Proteintech, #28733), anti-P-MEK1/2 (1:1000, Cell Signaling Technology, #9154), anti-α-Tubulin (1:2000, Proteintech, #66031), HRP-goat anti-rabbit IgG (1:2000, Proteintech, #SA00001-2), and HRP-goat anti-mouse IgG (1:2000, Proteintech, #SA00001-1).

### Cell viability assay

After we digested the PC cells (PANC-1 and MIA PaCa-2) down using trypsin, we counted the total number of cells using a cell counting plate. We then added a medium containing 5000 PC cells to each well of a 96-well plate. The Cell Counting Kit-8 (CCK-8) assay (Dojindo, Japan) was used to observe the proliferative capacity of the cells. At periodic intervals, we measured and recorded the cells' absorbance values at 450 nm.

### Colony formation experiment

In this experiment, we adjusted the number of treated PC cells to 1 × 10^3^, gently resuspended them in media, added them into each well of a 6-well plate, and incubated them in the incubator for approximately two weeks. Subsequently, the cells were subjected to fixation using a 4% paraformaldehyde solution, followed by staining with crystal violet at a concentration of 0.25%. The colonies were counted and photographed.

### EdU assay

A 12-well plate was utilized to inoculate each well with a cell count of 2 × 10^4^, after which the cells proliferated until they reached an appropriate density. The cells were incubated for 2 h after introducing a 20 µM EdU stock solution. The cells were then secured with 4% paraformaldehyde solution. Fluorescence staining was carried out utilizing DAPI and the fluorescent dye iF555, following the manufacturer's specified guidelines. We used fluorescence microscopy (Nikon, Tokyo, Japan) to detect the EdU-positive rate in each interface.

### Test for wound healing

We first inoculated the appropriate PANC-1 cells, or MIA PaCa-2 cells, into 6-well plates. When the cell confluence reached 90–100% in each well, we scraped the cell layer with a 200 µl pipette tip at the appropriate position with the same force and speed to generate scratches. Generally, we choose three time points, e.g., 0 h, 24 h, and 48 h. Measure and compare the scratch area at these three time points. We assessed cell migration by measuring the scratch area and changes over time.

### Migration and invasion experiments

The Transwell apparatus (CoStar, USA) was prepared. Two hundred microliters of serum-free medium (inoculated with 1 × 10^4^ cells) were added to the Transwell upper chamber (with or without Matrigel gel), and 800 µl of medium with 10% FBS was added to the lower chamber. Cells were incubated for 24–36 h in a 37 °C incubator with 5% CO_2_. Following a 36-h incubation period, the cells underwent two washes with phosphate-buffered saline (PBS). Last, we collected the cells and stained them so that the cells could be visualized for comparison.

### Immunohistochemistry (IHC)

After obtaining the paraffin-embedded sections, we deparaffinized and hydrated them. The tissue sections were then sealed to reduce endogenous peroxidase activity. Antigen repair was then performed on the tissues. Afterward, the tissues were incubated with PYGB antibody at 4 °C overnight. The tissues were then incubated accordingly with secondary antibodies for a suitable time. The tissues were color-built with DAB and eventually restained, then instantly microscopically observed and photographed.

### In vivo experimentation

First, we acquired 6- to 7-week-old female BALB/c nude mice from Collective Pharmachem. The rearing environment was maintained at a temperature of 22 ± 1 °C, a relative humidity of 50 ± 1%, and a light/dark cycle of 12/12 h. The mice were subjected to subcutaneous injection of 2 × 10^6^ cells in the right axilla and randomly allocated into five groups, each consisting of five mice. Tumor volume was measured at regular intervals using Vernier calipers and then estimated using the formula (length times width squared divided by 2). After seven weeks, the nude mice were subjected to euthanasia, and then the tumors were extracted, weighed, and photographed. The cell density was adjusted to 1 × 10^6^ in the liver metastasis model. Subsequently, nude mice were imaged using an in vivo bioluminescence imaging system (IVIS^®^Lumina III) to capture liver metastases in nude mice. The animal investigations, which encompassed procedures such as mouse euthanasia, were carried out in strict adherence to the norms and criteria set forth by Guizhou Medical University for the care of animals inside their institution.

### Statistical analysis

GraphPad Prism 8.0 or SPSS 25.0 statistical software was used to analyze the data. Measurements conforming to a normal distribution are expressed as the mean ± standard deviation. Comparisons between the two groups were made using the *t* test, *t*' test, or Wilcoxon test. Paired samples were compared using the paired samples *t* test. Comparisons of more than three groups were made using Dunnett’s *t* test and ANOVA. Count information was analyzed by applying the Mann‒Whitney *U* test. *P* < 0.05 was considered to indicate statistical significance.

## Result

### Screening critical genes related to glucose metabolism in PC

**Fig. 2:**
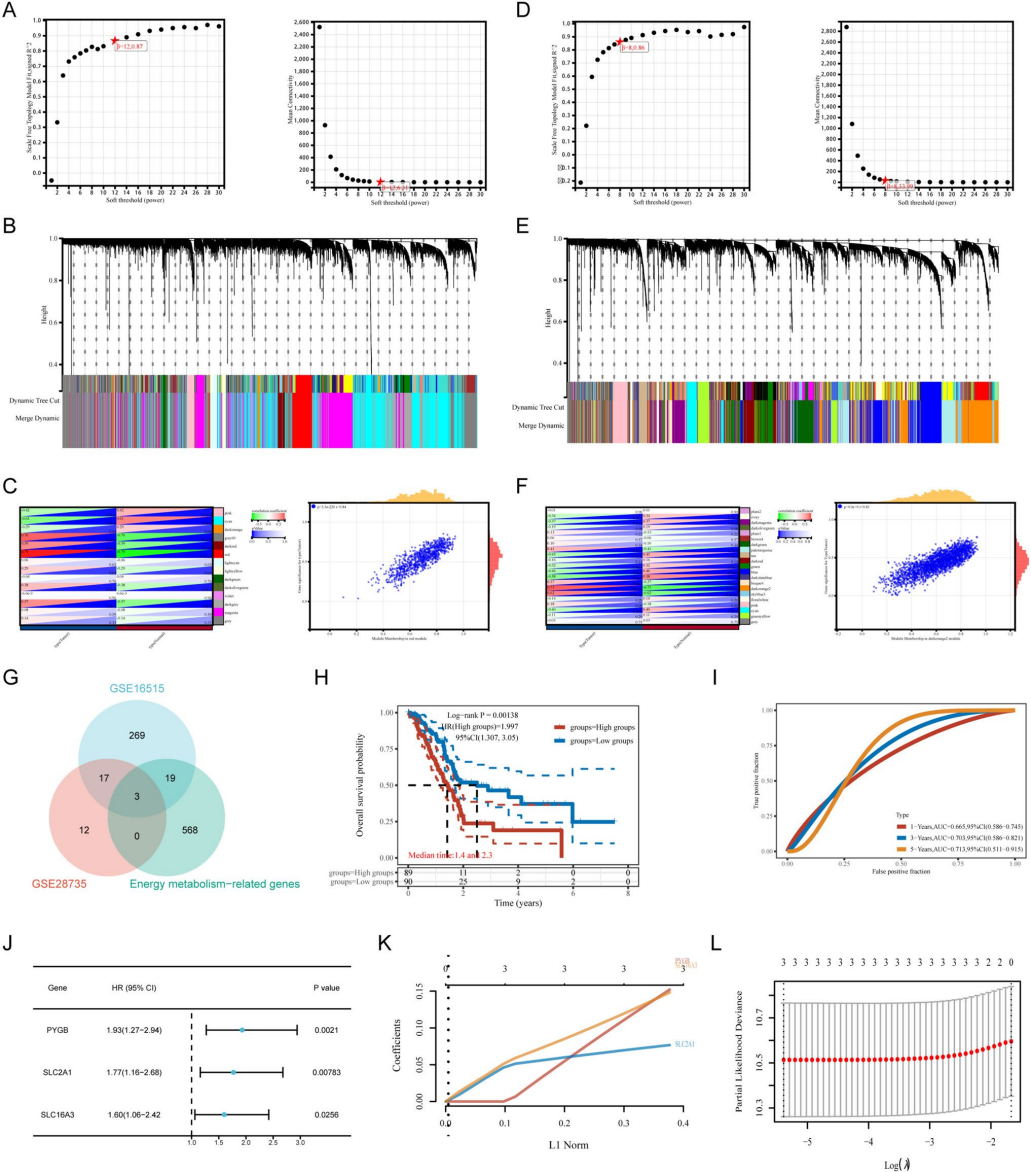
Construction of a hierarchical clustering tree and modeling of risk prognosis. **A**–**C** Select appropriate soft thresholds in the GSE16515 dataset and perform average connectivity analysis for 1–30 soft-threshold powers. After creating a hierarchical clustering tree, correlation heatmaps and scatter plots show that the RED module strongly correlates with tumors. **D**–**F** Hierarchical clustering trees were created in the GSE16515 dataset in the same way as above. The DARKORANGE2 module had the strongest correlation with tumors. **G** Taking the intersection of the RED module, the DARKORANGE2 module, and the energy metabolism-related genes module, we found that PYGB, SCL2A1, and SLC16A3 are vital genes in PC. **h**–**J** The model consisting of these three genes was verified in the TCGA database to have a strong correlation with both diagnosis and prognosis of PC and is a risk factor for poor prognosis of PC. **K** Lasso coefficient profiles of the three PC prognostic genes. **L** Riskscore = (0.152) × PYGB + (0.0767) × SLC2A1 + (0.1482) × SLC16A3 for the three prognostic genes obtained using tenfold cross-validated lasso regression using minimum λ

To screen critical genes related to glucose metabolism in PC, we constructed WGCNA co-expression networks for the GSE16515 dataset (Fig. 2A–C) and the GSE28735 dataset (Fig. 2D–F), respectively. First, to construct the scale-free network, we set the optimal soft-threshold power (*β*) of the GSE16515 dataset and the GSE28735 dataset to 12 (Fig. 2A) and 8 (Fig. 2D), respectively. Then, we introduced genes with similar expression patterns into the same module by a dynamic tree-cutting algorithm (module size = 30) to form a hierarchical clustering tree of different modules. In GSE16515, we obtained a total of 14 gene modules, among which the most relevant module to PC was red (*r* = 0.84, *P* < 0.05) (Fig. 2C). In GSE28735, we obtained a total of 21 gene modules, among which the most relevant module to PC was darkorange2 (*r* = 0.81, *P* < 0.05) (Fig. 2F). Next, we downloaded 590 genes related to energy metabolism from the GSEA database. We took the intersection with hub genes in the RED and DARKORANGE2 modules to find critical genes related to energy metabolism in PC (Fig. 2G). We found that PYGB, SCL2A1, and SLC16A3 were essential genes affecting the prognosis of PC (Fig. 2G). In addition, based on the TCGA database we found that the gene module consisting of PYGB, SCL2A1 and SLC16A3 had a significant impact on the diagnosis and prognosis of PC (Fig. 2H, I) and was a risk factor for poor prognosis of PC (Fig. 2J). We included them all in the LASSO model to reduce multicollinearity. Based on the LASSO model, we can infer a prognostic risk score for the feature, Riskscore = (0.152) × PYGB + (0.0767) × SLC2A1 + (0.1482) × SLC16A3 (Fig. 2K, L). The results showed that PYGB, SCL2A1, and SLC16A3 were all prognostic risk factors for PC, but PYGB had the most significant impact on the prognosis of PC. Next, we further used pan-cancer analysis to understand the effects of PYGB in tumors.

### Pan-cancer analysis of PYGB

**Fig. 3:**
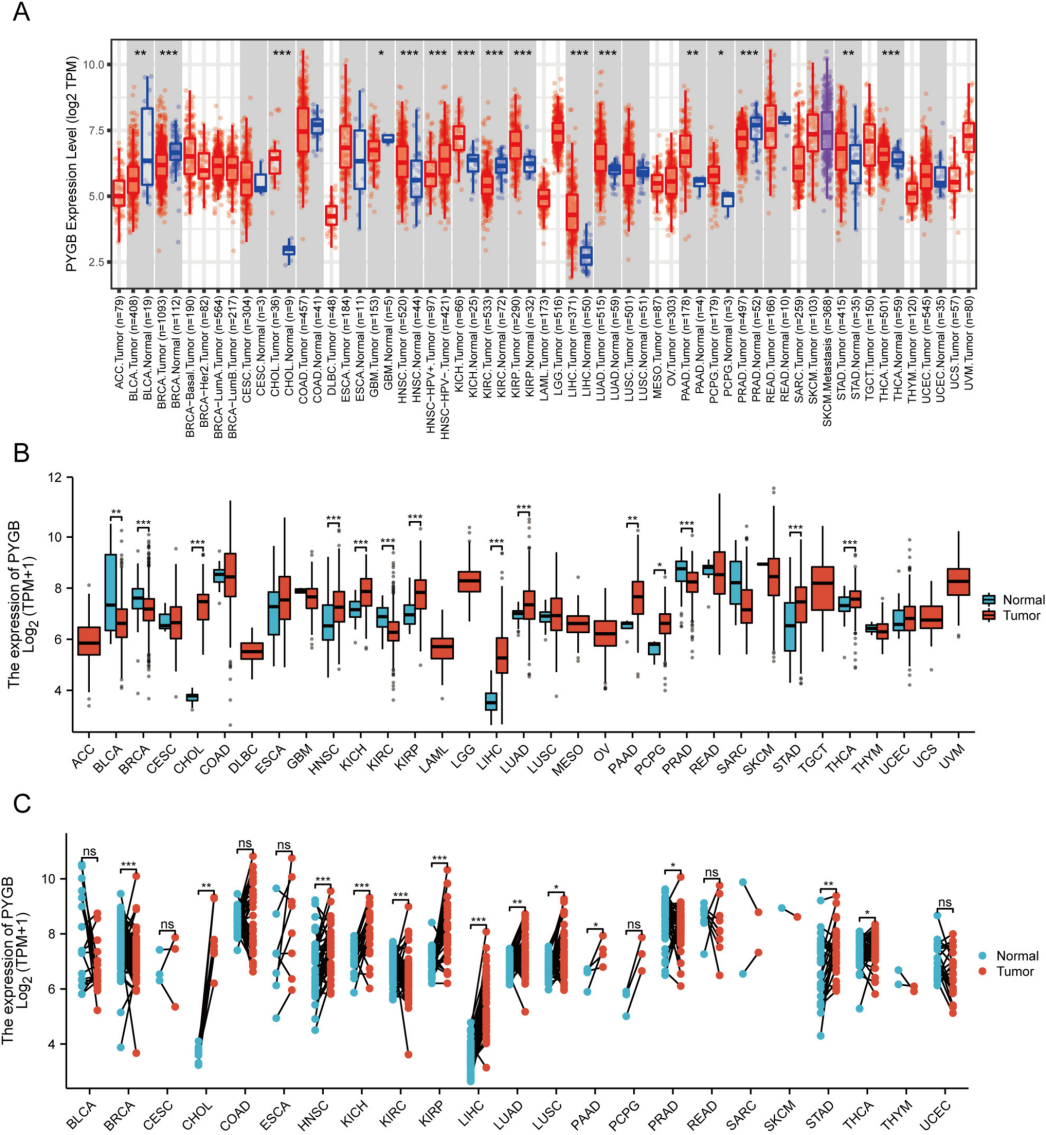
Expression levels of PYGB in pan-cancer tissues. **A** Expression levels of PYGB in pan-cancer tissues in the TIMER 2.0 database. **B** TCGA and GTEx data show different expressions of PYGB in pan-tumors. **C** TCGA dataset pairing PYGB expression in cancer tissues and adjacent normal tissues

**Fig. 4:**
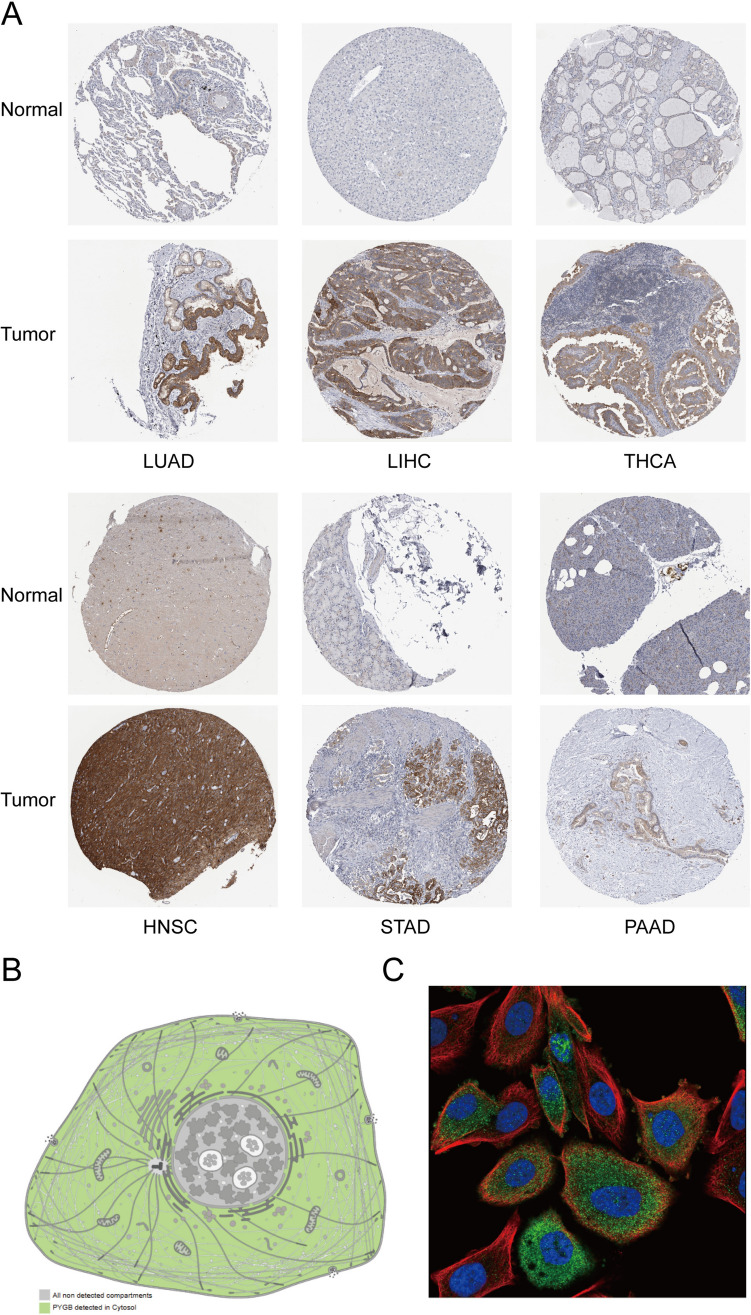
PYGB protein expression levels and subcellular localization based on the HPA database. **A** Immunohistochemical images of protein expression of PYGB in LUAD, LIHC, THCA, HNSC, STAD, and PAAD; **B** and **C** mock-ups and immunofluorescence maps of subcellular localization of PYGB

**Fig. 5:**
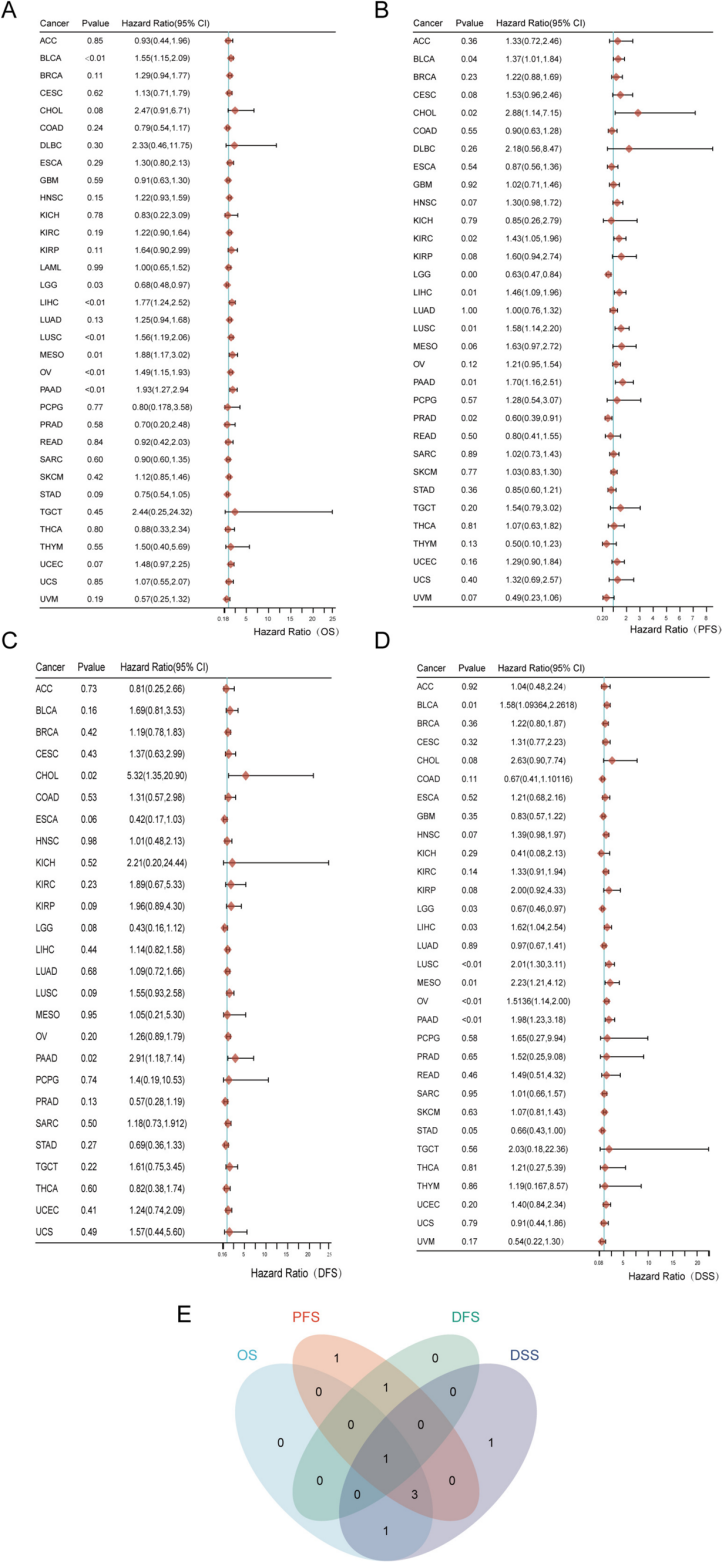
Prognostic value of PYGB in pan-cancer. **A**–**D** The results of OS, DFS, DFS and DSS are presented as forest plots. **E** Intersection analysis of the effect of PYGB on OS, DFS, DFS and DSS in pan-cancer

To determine the potential role of PYGB in human cancers, we first examined the expression levels of the PYGB gene in pan-cancer using the TIMER 2.0 database. We found that the mRNA level of PYGB was significantly elevated in tumor vs. normal tissues for most malignant tumors (Fig. 3A). Since the TCGA database lacked normal samples, we included the GTEx database to supplement the information on normal tissues. The results showed that high expression of PYGB was present in, including BLCA, BRCA, CHOL, HNSC, KICH, KIRC, KIRP, LIHC, LUAD, PAAD, PCPG, PRAD, STAD, and THCA (Fig. 3B). In addition, we explored the expression level of PYGB in matched samples and obtained similar results (Fig. 3C). When the expression levels of PYGB in LUAD, LIHC, THCA, HNSC, STAD, and PAAD were further compared with those in the corresponding normal tissues in the HPA database (Fig. 4A), it was observed that PYGB protein levels were also increased in multiple tumors that were mentioned above. On the other hand, we queried PYGB's subcellular localization using the HPA database (Fig. 4B and C) and discovered that PYGB was chiefly localized in the cytoplasm. The above discoveries indicate that PYGB expression is dysregulated in many tumor types. Finally, the PFS, OS, DSS, and DFS correlation with PYGB expression was investigated in the pan-cancer dataset. The findings showed a significant correlation between poor prognosis and elevated PYGB expression in various tumor types (Fig. 5A–D). Overall, by comprehensively evaluating the four prognostic states, we observed that PYGB was most closely associated with patient prognosis in PC (Fig. 5E). We next concentrated on exploring the relationship between PC and the PYGB gene.

### Bioinformatics-based analysis of the relationship between PC and PYGB

**Fig. 6:**
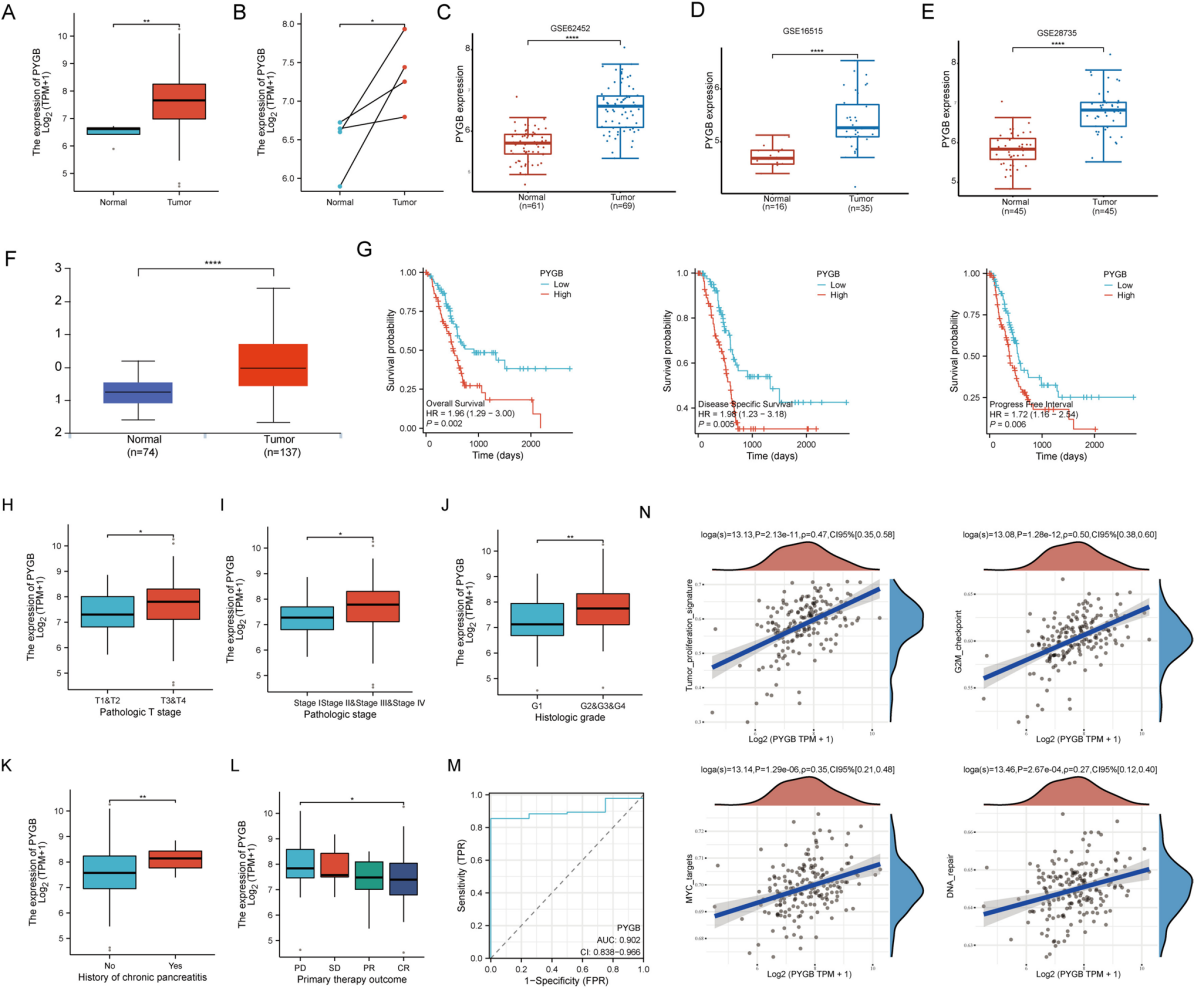
PC’s Prognosis and clinicopathologic features were connected to PYGB expression. **A**–**F** TCGA + GTEx, GEO, and UALCAN databases showed that PYGB was highly expressed in PC tissues. **G** High expression of PYGB indicates poorer OS, PFS, and PFI in patients. **H**–**L** High expression of PYGB is associated with multiple clinicopathologic parameters in PC patients. **M** ROC curves show a high accuracy rate for diagnosing PC. **N** The expression of PYGB exhibits a favorable correlation with various processes

To examine the expression of PYGB in PC, we discovered that the mRNA level of PYGB was significantly increased in PC in both the TCGA + GTEx and GEO datasets (Fig. 6A–E). Additionally, our investigation based on the UALCAN database revealed that the protein expression of PYGB was significantly upregulated in PC (Fig. 6F). We found that elevated PYGB gene expression was associated with unfavorable OS, DSS, and PFI in PC patients (Fig. 6G). Moreover, the higher the expression of PYGB was, the worse the clinical stage and pathologic grading of PC patients (Fig. 6H–K) and the worse the efficacy assessment (Fig. 6L). The upregulation of PYGB gene expression has been linked with an elevated susceptibility to PC, making it a dependable diagnostic biomarker (Fig. 6M). COX regression analysis based on the TCGA database likewise confirmed PYGB impact on PC patient prognosis (supplementary Table 2). We also found that high PYGB gene expression is closely associated with many tumor-related biological processes (Fig. 6N). We hypothesized that PYGB is a valuable oncogenic factor in PC.

### PYGB is highly expressed in PC cells and tissues

**Fig. 7:**
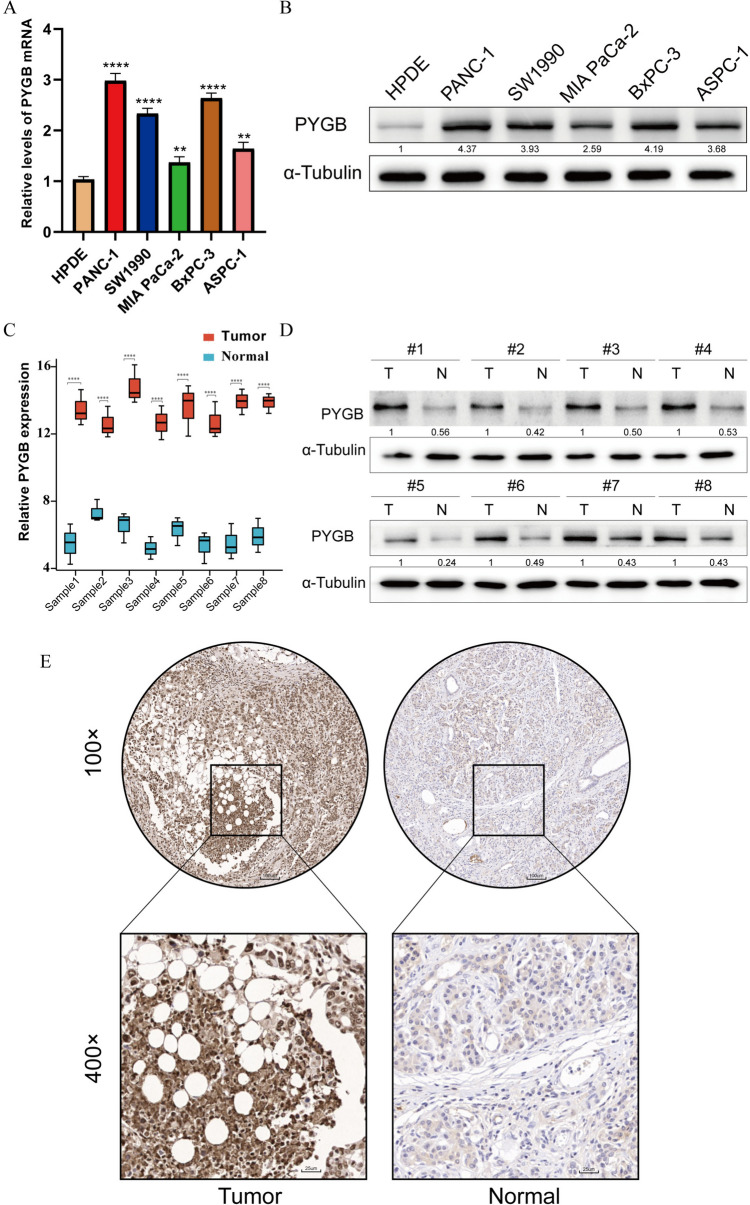
Expression of PYGB in PC cells and tissues. **A** and **B** PC cell lines express high levels of PYGB mRNA and protein. **C**–**E** In PC tissues, PYGB mRNA and protein levels are high

To determine PC PYGB expression, we first verified the expression level of the gene in PC cell lines. The findings showed that the expression of PYGB was dramatically augmented in several PC cell lines compared to HPDE6-C7 cells. MIA PaCa-2 cells displayed the lowest PYGB expression level within the cell lines under investigation, while PANC-1 demonstrated the highest (Fig. 7A and B). Therefore, we used MIA PaCa-2 and PANC-1 cells for subsequent studies. In addition, by examining 8 PC tissues and paired paracancerous tissues, we also found that both the transcript and protein levels of PYGB were highly expressed in PC tissues (Fig. 7C and D), and immunohistochemistry experiments further confirmed this result (Fig. 7E).

### PYGB promotes the proliferation, invasion, and metastasis of PC

**Fig. 8:**
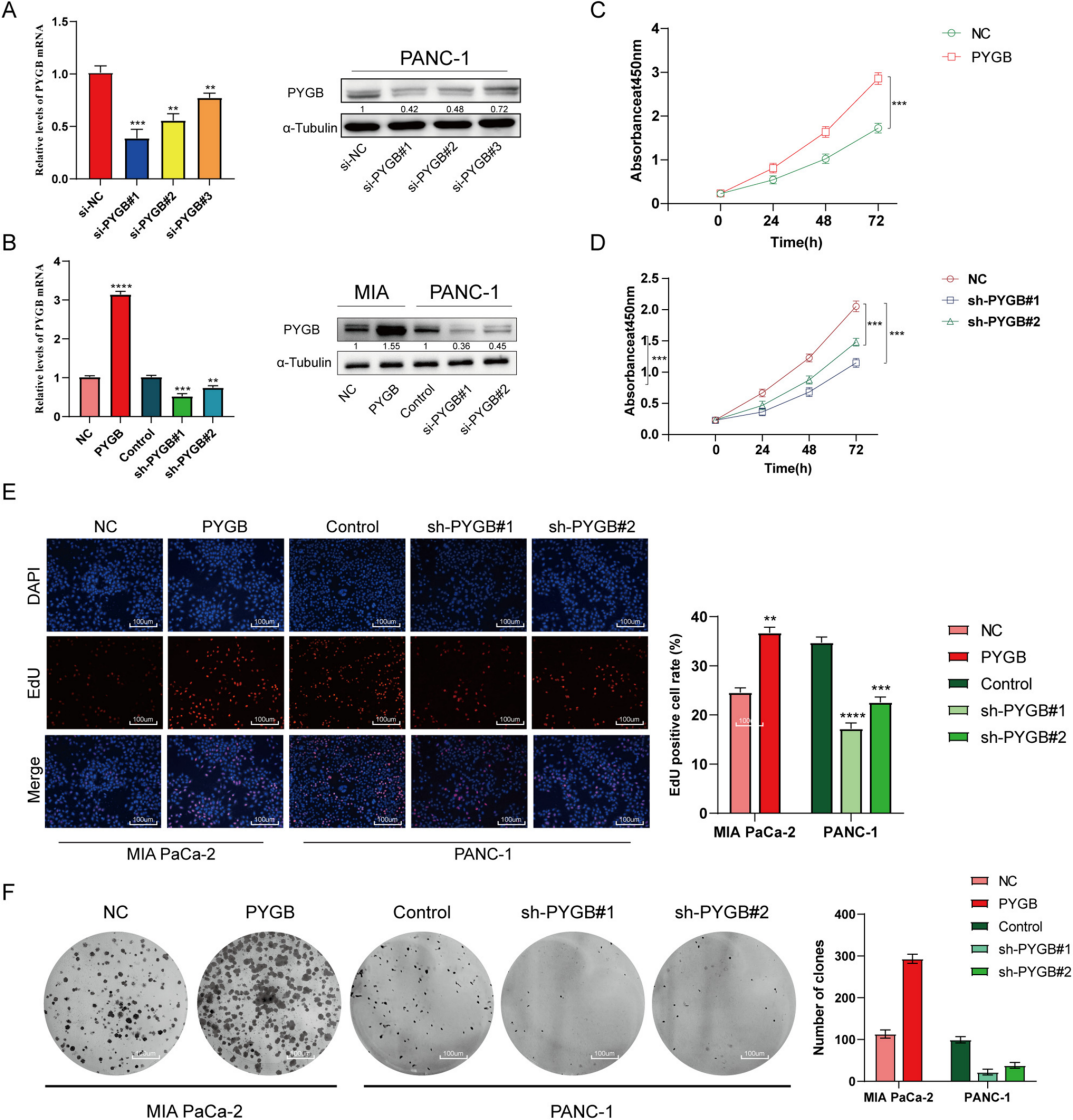
PYGB promotes the proliferative capacity of PC cells. **A** Validation of the transfection efficiency of small interfering RNA. **B** Detection of the transfection efficiency for lentiviruses for the upregulation/downregulation of PYGB. **C**–**F** The effect of PYGB gene expression on PC cell proliferation was investigated using EdU, colony formation, and CCK-8 assays

**Fig. 9:**
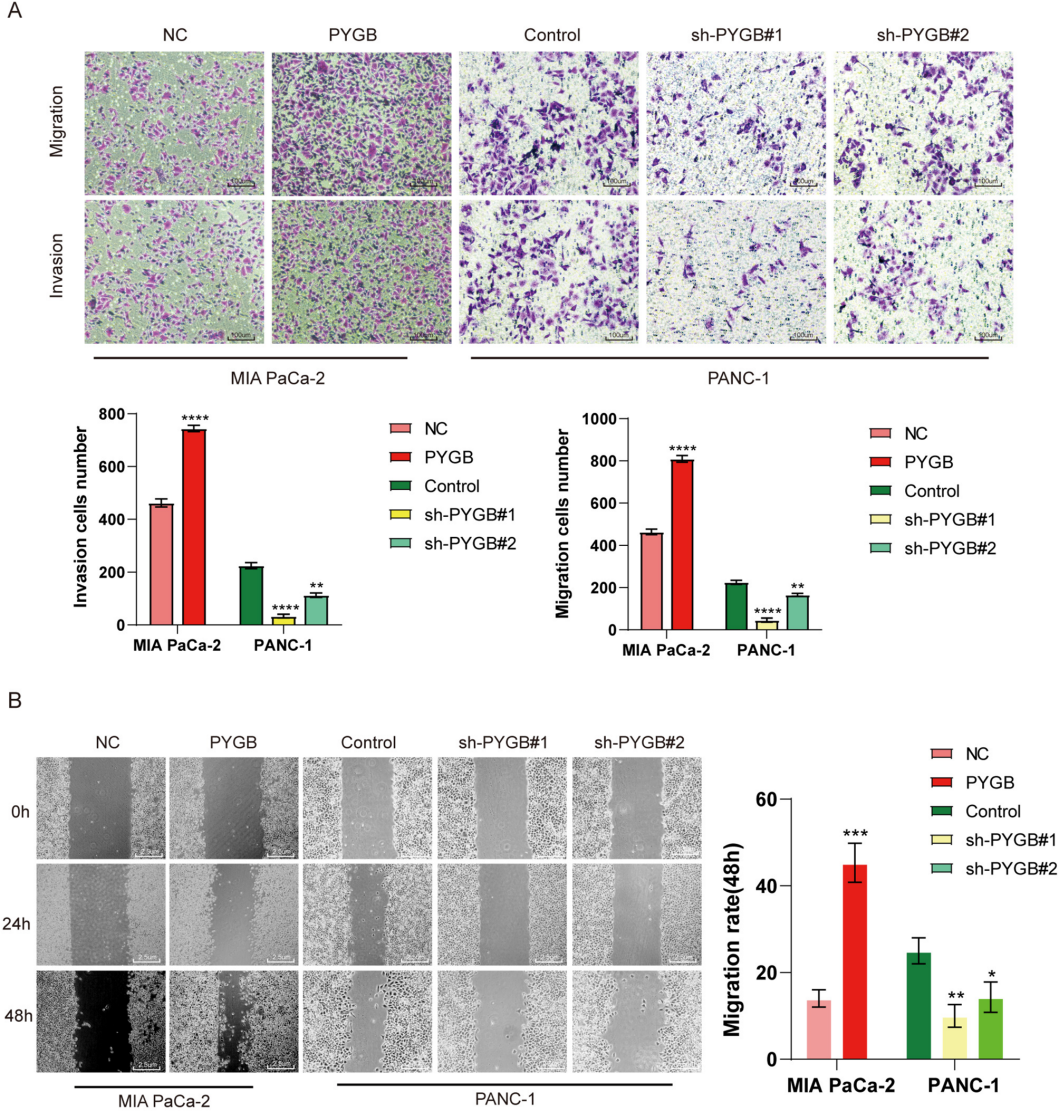
PYGB promotes the metastatic ability and invasion of PC cells. **A** and **B** Transwell and wound healing assays were employed to assess the impact of PYGB gene expression on PC cells' invasive and metastatic potential

**Fig. 10:**
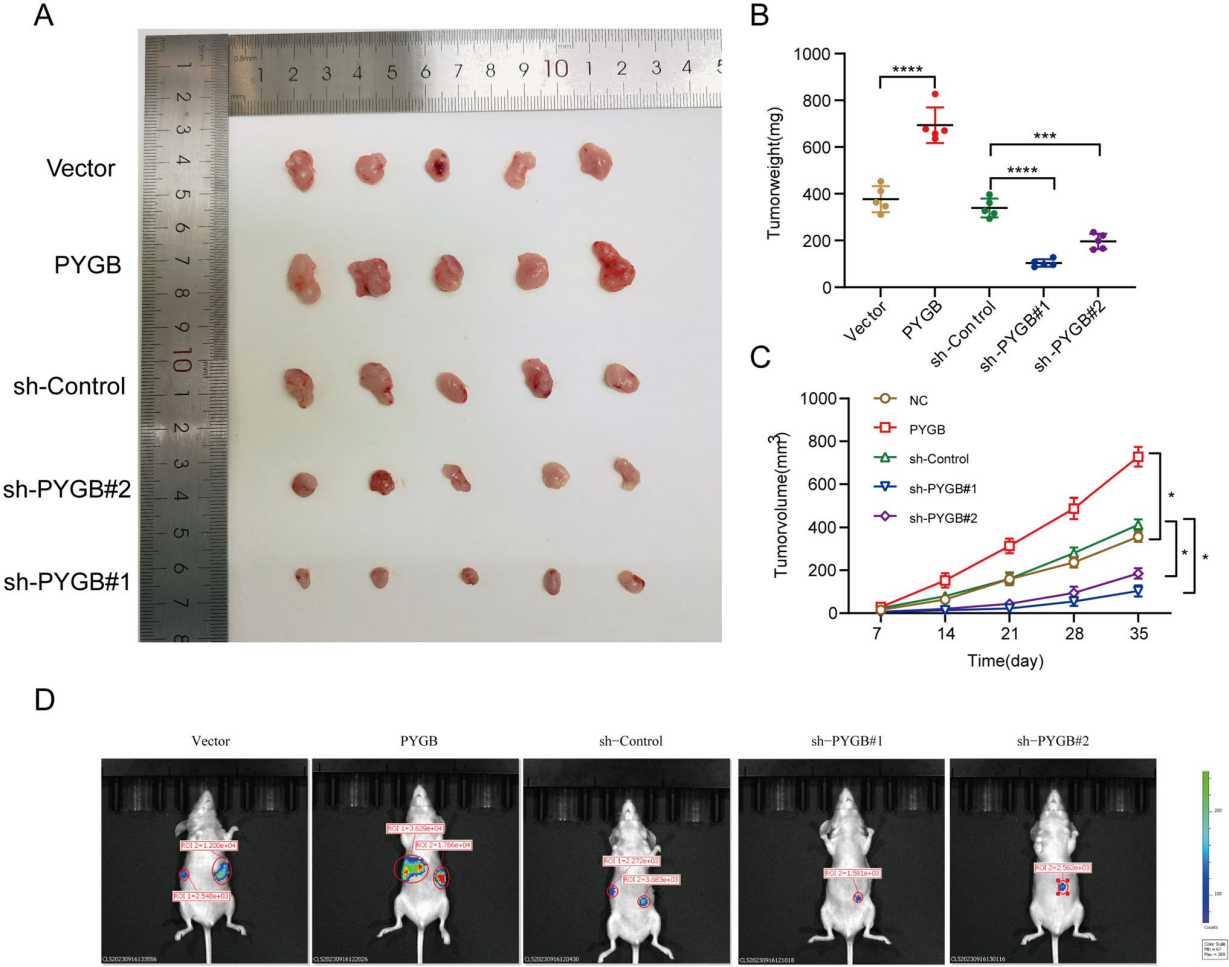
In vivo experiments confirmed that PYGB promotes the proliferation of PC cells. **A**–**C** Subcutaneous tumor formation experiments in nude mice revealed the effects of PYGB gene regulation on tumor size and weight. **D** The effect of the PYGB gene on the formation of liver metastases was observed by constructing a liver metastasis model

Three small interfering RNAs (siRNAs) were initially devised to manipulate PYGB expression in PC cells to investigate the impact of PYGB on PC. As shown in Fig. 8A, the findings showed that the si-PYGB#2 sequences and si-PYGB#1 exhibited the best silencing effect. We subsequently generated lentiviral vectors capable of inducing stable upregulation or downregulation in target cells. The infection effectiveness of these vectors was then assessed and validated (Fig. 8B). Through CCK-8 experiments, and we found that PC cells overexpressing PYGB had a significantly faster proliferation rate, a significantly higher EdU positivity rate, and a higher clonal island formation ability than controls. In contrast, these abilities were significantly reduced after PYGB knockdown (Fig. 8C–F). Indeed, by applying the scratch assay and the Transwell assay, we concluded that overexpression of PYGB in PC cells significantly enhances their invasive and migratory abilities. In contrast, PYGB knockdown significantly reduced both abilities (Fig. 9A and B). To conduct a more comprehensive examination of the involvement of PYGB in PC, subcutaneous tumor formation experiments were performed in nude mice to observe the effect of PYGB expression on the proliferative capacity of PC cells. The study revealed a significant increase in tumor weight and volume in nude mice following PYGB upregulation compared to the control group (Fig. 10A–C). Conversely, the opposite effect was observed when PYGB was downregulated. Moreover, suspensions of PC cells with stable PYGB overexpression were implanted in the spleens of nude mice to construct a liver metastasis model. In the overexpression group, there was a significant increase in the number and size of liver metastases compared to that in the control group. On the other hand, liver metastasis was significantly suppressed in nude mice injected with PC cells with knockdown of the PYGB gene (Fig. 10D). Overall, we conducted a series of in vitro and in vivo tests to investigate the impact of PYGB overexpression on the proliferation, invasion, and metastasis of PC cells. Our findings indicate that PYGB overexpression significantly enhances these cellular processes in PC cells.

### The PYGB-MAPK/ERK axis promotes PC progression

**Fig. 11:**
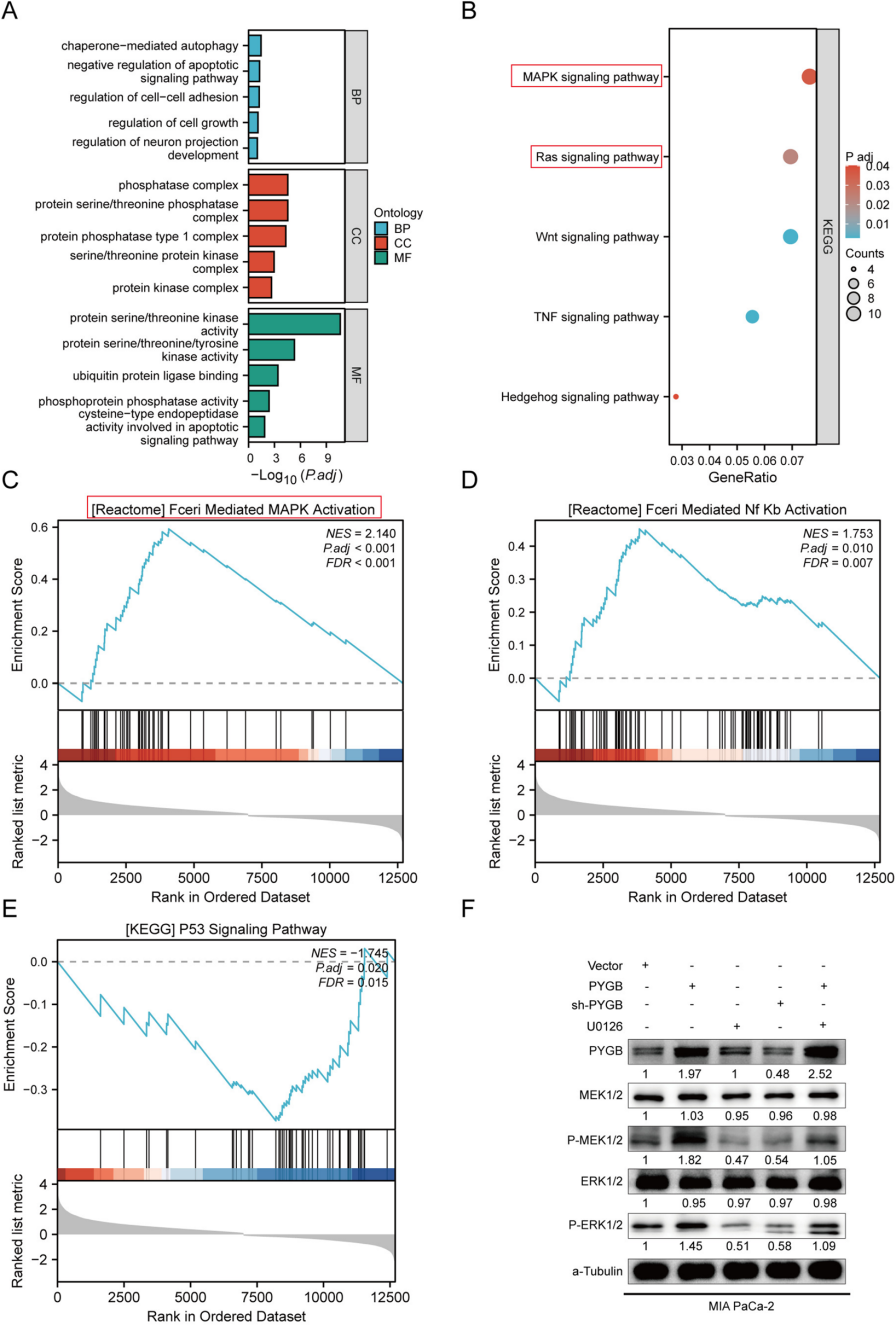
PYGB regulates the MAPK/ERK signaling pathway. **A** and **B** GO and KEGG studies revealed that PYGB exhibited associations with many metabolic pathways and signaling pathways implicated in tumorigenesis. **C**–**E** GSEA showed that PYGB was associated with multiple critical tumor-related signaling pathways, most notably the MAPK signaling pathway. **F** PYGB overexpression resulted in enhanced MEK and ERK phosphorylation

**Fig. 12:**
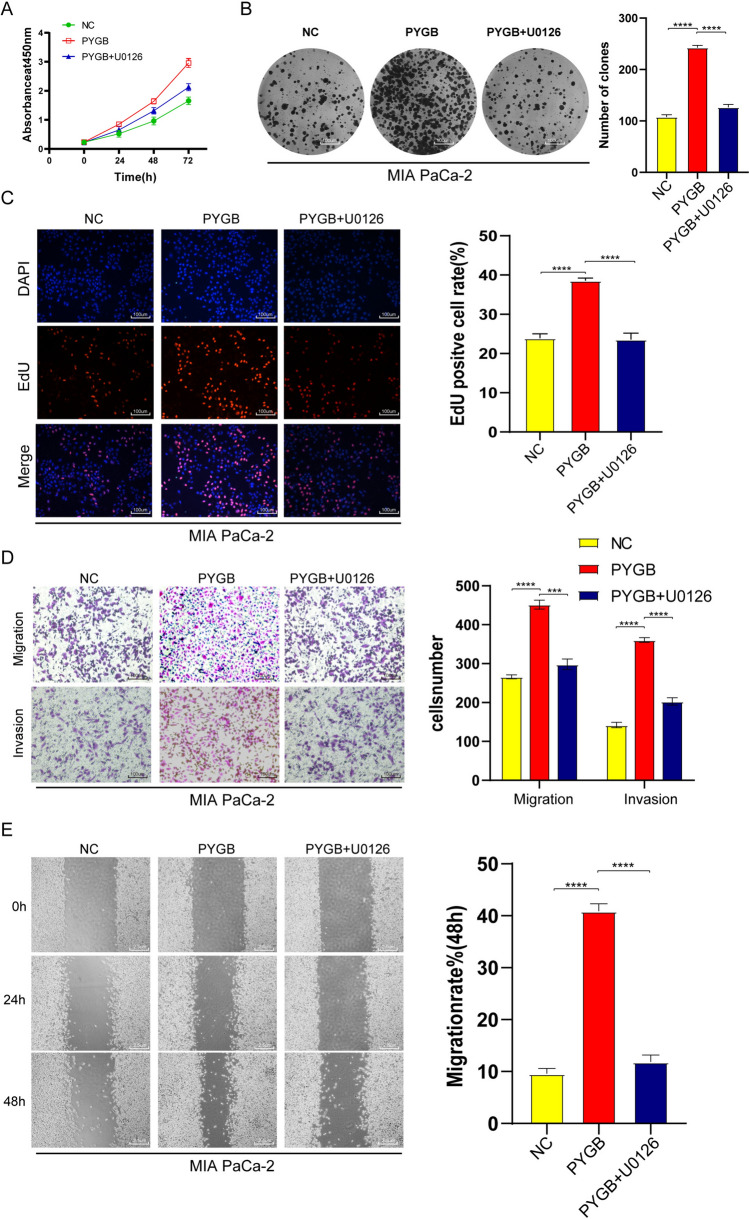
U0126 inhibited the promotion of PYGB in PC. **A**–**C** Colony formation, EdU, and CCK-8 assays demonstrated that U0126 blocked the proliferative ability of PYGB in PC cells. **D** and **E** The results of the scratch assay and Transwell assay showed that U0126 successfully inhibited the invasive and metastatic properties conferred by PYGB in PC cells

PYGB-associated gene enrichment was analyzed to determine the mechanism of PYGB carcinogenesis. In GO analysis, we found that PYGB genes were associated with pathways such as cell autophagy, apoptosis, cell adhesion, cell growth, serine/threonine protein kinase activity and ubiquitin-protein ligase (Fig. 11A). Through the utilization of KEGG analysis, it was shown that the PYGB gene had associations with many signaling pathways, including but not limited to the MAPK signaling pathway, Ras pathway, Wnt route, Hedgehog pathway, and several others (Fig. 11B). By GSEA, we found that PYGB positively regulated the MAPK pathway and NF-κB pathway and negatively regulated the P53 pathway (Fig. 11C–E). The Ras/Raf/MEK/ERK signaling pathway facilitates PC cell proliferation, invasion, and metastasis (Shin et al. 2020). Given that both KEGG analysis and GSEA showed high enrichment of the MAPK and Ras pathway, we conducted additional experimental investigations to validate the association between PYGB and the MAPK/ERK signaling pathway. The total protein levels of MEK and ERK were unaltered after the overexpression of PYGB. In contrast, the levels of phosphorylated MEK and ERK exhibited a substantial increase, and the administration of the MEK inhibitor U0126 effectively counteracted this effect (Fig. 11F). Further rescue experiments also showed that PYGB promoted PC cell proliferation, invasion, and metastasis. However, the MEK inhibitor U0126 inhibited this process (Fig. 12A–E). Therefore, we concluded that PYGB promotes PC proliferation, invasion, and metastasis by activating the MAPK/ERK signaling pathway.

## Discussion

PC is a very aggressive tumor marked by rapid progression, so patients are often diagnosed in the advanced stage (Park et al. 2021). Although encouraging research has revealed diagnostic and treatment-related biomarkers and drugs, the overall biological mechanisms of PC remain to be discovered (Sherman and Beatty 2023).

Glycogen is a branched-chain polysaccharide made from glucose, a type of stored energy in animals (Zois and Harris 2016). Glycogen levels have been reported to be negatively correlated with the proliferation rate of tumor cells; for instance, during the metabolism of glycogen, PYG catalyzes the cleavage of glycogen and its conversion to glucose-6-phosphate (Marr et al. 2022), which can take part in the glycolysis process of neoplastic cells to provide energy for cancer progression (Cao et al. 2020). Additionally, an expanding body of evidence indicates that glycogen metabolism exhibits intricate interactions with numerous pivotal signaling pathways, hence exerting a significant influence on the processes of tumor proliferation, invasion, and metastasis (Shorthouse et al. 2022; Yan et al. 2019), and one possible anticancer strategy is to suppress glycogen phosphorylase synthesis, thereby suppressing glycogen metabolism to interrupt the energy supply to tumors (Dauer and Lengyel 2019; Favaro and Harris 2013). Favaro reported that glucose utilization via PYGL sustains cancer cell proliferation and prevents premature senescence (Favaro et al. 2012). In addition, PYGL-mediated reprogramming of glucose metabolism promotes PC EMT and metastasis (Ji et al. 2023). It has also been shown that hypoxia-inducible factors can induce glycogen synthesis under hypoxic conditions and promote cancer cell survival (Pelletier et al. 2012). PYGM can also be a biomarker for head and neck squamous cell carcinoma (Jin and Yang 2019). PYGB is the other isoenzyme of PYG in humans, and it cleaves glycogen to G6P, which supplies an energy source to neoplastic cells under nutrient-deficient conditions (Rich et al. 2009). Recent evidence suggests that PYGB is involved in the tumorigenesis of many cancers. For example, PYGB promotes the proliferation and migration of non-small cell lung and gastric cancer by activating the Wnt pathway (Xiao et al. 2020). Prostate cancer cell growth is suppressed by PYGB inhibition through the NF-κB pathway (Wang et al. 2018). However, the cancer-promoting mechanism and expression level of PYGB in cells or PC tissues have not been explored. Through a series of bioinformatics analyses, we found that PYGB has a potential oncogenic role in various tumors. Notably, both the transcript and protein levels of PYGB were highly expressed in PC, causing poorer OS, DFS, DSS, and PFI in patients, which is uniquely valuable to study in PC. In addition, we found that high expression of the PYGB gene was associated with the G2/M cell cycle checkpoint, cancer cell proliferation, DNA damage repair, and MYC gene expression. Interestingly, we found that the protein and mRNA levels of PYGB were high in tissues and PC cells by histological and cytological validation experiments. Such results prompted us to consider that PYGB promotes PC progression, especially proliferation, invasion, and metastasis. We then showed through a series of in vitro and in vivo experiments that PYGB promoted PC cell proliferation, invasion, and metastasis. We thus recommend that PYGB may serve as a promising gene therapy target and a novel diagnostic biomarker for PC.

The involvement of various signaling pathways, including the MAPK signaling pathway, the PI3K/AKT pathway, the Hedgehog signaling pathway, and the Notch signaling pathway, in the malignant progression of PC is widely recognized (Zhou et al. 2023). The MAPK signaling pathway holds significant importance within the eukaryotic signaling network, serving as a crucial conduit for several cellular processes, such as cell proliferation, differentiation, apoptosis, and stress responses (Wagner and Nebreda 2009). MAPK is a group of evolutionarily conserved serine/threonine kinases divided into four subfamilies: ERK, p38, JNK, and BMK1, representing the four classical MAPK pathways (Lee et al. 2020). As one of the classical pathways in the MAPK/ERK signaling pathway, upstream signaling molecules, such as RAS proteins, activate downstream RAF proteins, which activate ERK by phosphorylating MEK proteins, which activate ERK. Phosphorylated ERK translocates into the nucleus, phosphorylates genes, such as c-Jun, c-Fos, or other genes, and activates a variety of transcription factors to induce the expression of genes, such as Cyclin D1, E2F1, Ki67, and Bcl-2, and to inhibit the expression of genes, such as P27 and Bad (Zhang et al. 2022). Targeting glucose metabolism sensitizes PC to MEK inhibitors (Yan et al. 2021). According to different enrichment analysis methodologies, we found that PYGB promotes cancer by acting as a link between various oncogenic pathways. Given that both KEGG analysis and GSEA showed high enrichment of the MAPK and Ras pathways, we examined the regulatory role of PYGB in the MAPK/ERK signaling pathway. PYGB overexpression promoted the phosphorylation of ERK1/2 and MEK1/2. Moreover, the promoting effects of PYGB on PC cell proliferation, invasion, and metastasis were blocked by the MEK inhibitor U0126. These results indicate that PYGB may facilitate PC malignant progression potentially via the MAPK/ERK signaling pathway.

Although this study explored the value of PYGB in PC, there are some shortcomings. First, we conducted the study based on bioinformatics analysis rather than high-throughput sequencing, so the results need to be further validated. Next, although we measured the expression level of PYGB using clinical samples, the relationship with patient prognosis was not additionally investigated. Additionally, the study of PYGB in glycogen metabolism, glycolysis, and PC still needs to be addressed. Therefore, our future research direction is to explore the precancer mechanism of PYGB in PC further by assessing its role in glycogen metabolism.

In summary, we screened PYGB, an essential gene in PC, by WGCNA and LASSO regression and analyzed its research value in tumors. Then, we evaluated PYGB expression levels in PC and examined how PYGB promotes invasion, metastasis, and proliferation in PC. In addition, we discovered that PYGB may promote the progression of PC malignancy by activating the MAPK/ERK signaling pathway. These results suggest that PYGB may be a new diagnostic biomarker and potential gene therapy target for PC.

### Supplementary Information

Below is the link to the electronic supplementary material.

Supplementary file1 (DOCX 13 KB)

Supplementary file2 (DOCX 15 KB)

## Data Availability

The data sets generated during and/or analyzed during the current study are available from the corresponding author on reasonable request.

## References

[CR115] Barrett T, Wilhite SE, Ledoux P, Evangelista C, Kim IF, Tomashevsky M, Marshall KA, Phillippy KH, Sherman PM, Holko M, Yefanov A, Lee H, Zhang N, Robertson CL, Serova N, Davis S, Soboleva A (2013). NCBI GEO: archive for functional genomics data sets–update. Nucleic Acids Res.

[CR1] Bryant KA-O, Stalnecker CA-O, Zeitouni D, Klomp JE, Peng S, Tikunov AP (2019). Combination of ERK and autophagy inhibition as a treatment approach for pancreatic cancer. Nat Med.

[CR2] Cao L, Wu J, Qu X, Sheng J, Cui M, Liu S (2020). Glycometabolic rearrangements–aerobic glycolysis in pancreatic cancer: causes, characteristics and clinical applications. J Exp Clin Cancer Res CR.

[CR120] Chandrashekar DS, Karthikeyan SK, Korla PK, Patel H, Shovon AR, Athar M, Netto GJ, Qin ZS, Kumar S, Manne U, Creighton CJ, Varambally S (2022). UALCAN: An update to the integrated cancer data analysis platform. Neoplasia.

[CR107] Chrysina ED (2010). The prototype of glycogen phosphorylase. Mini Rev Med Chem.

[CR109] Chou JY, Jun HS, Mansfield BC (2010). Glycogen storage disease type I and G6Pase-β deficiency: etiology and therapy. Nat Rev Endocrinol.

[CR103] DeBerardinis RJ, Chandel NS (2020). We need to talk about the Warburg effect. Nat Metab.

[CR3] da Paixão VF, Sosa OJ, da Silva Pellegrina DV, Dazzani B, Corrêa TB, Risério Bertoldi E (2022). Annotation and functional characterization of long noncoding RNAs deregulated in pancreatic adenocarcinoma. Cell Oncol (dordr).

[CR4] Dauer P, Lengyel E (2019). New roles for glycogen in tumor progression. Trends in Cancer.

[CR5] Favaro E, Harris AL (2013). Targeting glycogen metabolism: a novel strategy to inhibit cancer cell growth?. Oncotarget.

[CR6] Favaro E, Bensaad K, Chong MG, Tennant DA, Ferguson DJP, Snell C, Steers G (2012). Glucose utilization via glycogen phosphorylase sustains proliferation and prevents premature senescence in cancer cells. Cell Metab.

[CR7] Ferraro G, Mozzicafreddo M, Ettari R, Corsi L, Monti MC (2022). A proteomic platform unveils the brain glycogen phosphorylase as a potential therapeutic target for glioblastoma multiforme. Int J Mol Sci.

[CR8] Guo YJ, Pan WW, Liu SB, Shen ZF, Xu Y, Hu LL (2020). ERK/MAPK signalling pathway and tumorigenesis. Exp Ther Med.

[CR9] Halbrook CJ, Lyssiotis CA, Pasca di Magliano M, Maitra A (2023). Pancreatic cancer: advances and challenges. Cell.

[CR105] Hindson J (2021). Glycogen phase separation and liver cancer. Gastroenterol Hepatol.

[CR10] Ji Q, Li H, Cai Z, Yuan X, Pu X, Huang Y (2023). PYGL-mediated glucose metabolism reprogramming promotes EMT phenotype and metastasis of pancreatic cancer. Int J Biol Sci.

[CR11] Jin Y, Yang YA-O (2019). Biosci Rep.

[CR12] Lee S, Rauch JA-O, Kolch WA-O (2020). Targeting MAPK Signaling in Cancer: Mechanisms of Drug Resistance and Sensitivity. Int J Mol Sci.

[CR118] Li T, Fan J, Wang B, Traugh N, Chen Q, Liu JS, Li B, Liu XS (2017). TIMER: a web server for comprehensive analysis of tumor-infiltrating immune cells. Cancer Res.

[CR13] Liu F, Yang X, Geng M, Huang M (2018). Targeting ERK, an Achilles' Heel of the MAPK pathway, in cancer therapy. Acta Pharm Sin B.

[CR110] Kim GY, Kwon JH, Cho JH, Zhang L, Mansfield BC, Chou JY (2017). Downregulation of pathways implicated in liver inflammation and tumorigenesis of glycogen storage disease type Ia mice receiving gene therapy. Human MolGenet.

[CR14] Marr LA-O, Biswas DA-O, Daly LA-O, Browning C, Vial SCM, Maskell DA-OX (2022). Mechanism of glycogen synthase inactivation and interaction with glycogenin. Nat Commun.

[CR15] Mathieu C, Li de la Sierra-Gallay I, Duval R, Xu X, Cocaign A, Léger T (2016). Insights into brain glycogen metabolism: the structure of human brain glycogen phosphorylase. J Biol Chem.

[CR101] Meng Y, Sun J, Zhang G, Yu T, Piao H (2023). Imaging glucose metabolism to reveal tumor progression. Front Physiol.

[CR111] Mutel E, Abdul-Wahed A, Ramamonjisoa N, Stefanutti A, Houberdon I, Cavassila S, Pilleul F, Beuf O, Gautier-Stein A, Penhoat A, Mithieux G, Rajas F (2011). Targeted deletion of liver glucose-6 phosphatase mimics glycogen storage disease type 1a including development of multiple adenomas. J Hepatol.

[CR16] Park W, Chawla A, O'Reilly EM (2021). Pancreatic cancer: a review. JAMA.

[CR102] Pavlova NN, Thompson CB (2016). The emerging hallmarks of cancer metabolism. Cell Metab.

[CR104] Pavlova NN, Zhu J, Thompson CB (2022). The hallmarks of cancer metabolism: still emerging. Cell Metab.

[CR17] Pelletier J, Bellot G, Gounon P, Lacas-Gervais S, Pouysségur J, Mazure NM (2012). Glycogen synthesis is induced in hypoxia by the hypoxia-inducible factor and promotes cancer cell survival. Front Oncol.

[CR112] Resaz R, Vanni C, Segalerba D, Sementa AR, Mastracci L, Grillo F, Murgia D, Bosco MC, Chou JY, Barbieri O, Varesio L, Eva A (2014). Development of hepatocellular adenomas and carcinomas in mice with liver-specific G6Pase-α deficiency. Dis Models Mech.

[CR18] Rich SS, Goodarzi MO, Palmer ND, Langefeld CD, Ziegler J, Haffner SM, Bryer-Ash M (2009). A genome-wide association scan for acute insulin response to glucose in Hispanic-Americans: the insulin resistance atherosclerosis family study (IRAS FS). Diabetologia.

[CR106] Roach PJ, Depaoli-Roach AA, Hurley TD, Tagliabracci VS (2012). Glycogenand its metabolism: some new developments and old themes. Biochem J.

[CR19] Rovida E, Tusa I (2022). Targeting MAPK in cancer 2.0. Int J Mol Sci.

[CR20] Sherman MH, Beatty GL (2023). Tumor microenvironment in pancreatic cancer pathogenesis and therapeutic resistance. Annu Rev Pathol.

[CR21] Shin M, Kim J, Lim SA, Kim J, Lee K (2020). Current insights into combination therapies with MAPK inhibitors and immune checkpoint blockade. Int J Mol Sci.

[CR22] Shorthouse D, Bradley J, Critchlow SE, Bendtsen C, Hall B (2022). Heterogeneity of the cancer cell line metabolic landscape. Mol Syst Biol.

[CR23] Siegel R, Miller K, Fuchs HE, Jemal A (2022). Cancer statistics, 2022. CA Cancer J Clin.

[CR116] Subramanian A, Tamayo P, Mootha VK, Mukherjee S, Ebert BL, Gillette MA, Paulovich A, Pomeroy SL, Golub TR, Lander ES, Mesirov JP (2005). Gene set enrichment analysis: a knowledge-basedapproach for interpreting genome-wide expression profiles. Proc Nat Acad Sci.

[CR24] Sung H, Ferlay J, Siegel R, Laversanne M, Soerjomataram I, Jemal A, Bray F (2021). Global cancer statistics 2020: GLOBOCAN estimates of incidence and mortality worldwide for 36 cancers in 185 countries. CA J Cancer Clin.

[CR25] Szklarczyk D, Kirsch R, Koutrouli M, Nastou K, Mehryary F, Hachilif R (2023). The STRING database in 2023: protein-protein association networks and functional enrichment analyses for any sequenced genome of interest. Nucleic Acids Res.

[CR117] Tomczak K, Czerwińska P, Wiznerowicz M (2015). The Cancer Genome Atlas (TCGA): an immeasurable source ofknowledge. Contemp Oncol/Współczesna Onkologia.

[CR119] Uhlen M, Zhang C, Lee S, Sjöstedt E, Fagerberg L, Bidkhori G, Benfeitas R, Arif M, Liu Z, Edfors F, Sanli K, Von Feilitzen K, Oksvold P, Lundberg E, Hober S, Nilsson P, Mattsson J, Schwenk JM, Brunnström H, Glimelius B, Sjoblom T, Edqvist PH, Djureinovic D, Micke P, Lindskog C, Mardinoglu A, Ponten F (2017). A pathology atlas of the human cancer transcriptome. Sci.

[CR26] Wagner EF, Nebreda AR (2009). Signal integration by JNK and p38 MAPK pathways in cancer development. Nat Rev Cancer.

[CR27] Wang Z, Han G, Liu Q, Zhang W, Wang J (2018). Silencing of PYGB suppresses growth and promotes the apoptosis of prostate cancer cells via the NF-κB/Nrf2 signaling pathway. Mol Med Rep.

[CR100] Walker-Samuel S, Ramasawmy R, Torrealdea F, Rega M, Rajkumar V, Johnson SP, Richardson S, Gonçalves M, Parkes HG, Årstad E, Thomas DL, Pedley RB, Lythgoe MF, Golay X (2013). In vivo imaging of glucose uptake and metabolism in tumors. Nature Medicine.

[CR113] Wilson LH, Cho JH, Estrella A, Smyth JA, Wu R, Chengsupanimit T, Brown LM, Weinstein DA, Lee YM (2019). Liver glycogen phosphorylase deficiency leads to profibrogenic phenotype in a murine model of glycogen storage disease type VI. Hepatol Commun.

[CR28] Xia C, Dong X, Li H, Cao M, Sun D, He S, Yang F (2022). Cancer statistics in China and United States, 2022: profiles, trends, and determinants. Chin Med J.

[CR29] Xiao L, Wang W, Huangfu Q, Tao H, Zhang J (2020). PYGB facilitates cell proliferation and invasiveness in non-small cell lung cancer by activating the Wnt-β-catenin signaling pathway. Biochem Cell Biol Biochimie Et Biologie Cellulaire.

[CR30] Yan L, Raj P, Yao W, Ying H (2019). Glucose metabolism in pancreatic cancer. Cancers.

[CR31] Yan L, Tu B, Yao JA-O, Gong J, Carugo AA-O, Bristow CA (2021). Targeting glucose metabolism sensitizes pancreatic cancer to MEK inhibition. Cancer Res.

[CR32] Zhang RA-O, Ma L, Wei YA-O, Wei KA-O, Song T, Du ZA-O, Feng ZA-O (2022). KIF22 promotes development of pancreatic cancer by regulating the MEK/ERK/P21 signaling axis. Biomed Res Int.

[CR33] Zhou Y, Jin Z, Wang C (2019). Glycogen phosphorylase B promotes ovarian cancer progression via Wnt/β-catenin signaling and is regulated by miR-133a-3p. Biomed Pharmacother.

[CR34] Zhou K, Liu Y, Yuan S, Zhou Z, Ji P, Huang Q (2023). Signalling in pancreatic cancer: from pathways to therapy. J Drug Target.

[CR35] Zois CE, Harris AL (2016). Glycogen metabolism has a key role in the cancer microenvironment and provides new targets for cancer therapy. J Mol Med (berl).

