# Supplementary material for: Unveiling the role of PYGB in pancreatic cancer: a novel diagnostic biomarker and gene therapy target
Source: J Cancer Res Clin Oncol. 2024 Mar 14;150(3):127. doi: 10.1007/s00432-024-05644-2 (PMC10940407; doi:10.1007/s00432-024-05644-2)
Supplement: Supplementary file 2 — Supplementary file2 (DOCX 15 KB) [file 432_2024_5644_MOESM2_ESM.docx]

# Supplementary table 2. TCGA cancer abbreviations and the corresponding cancer type

| Characteristics | Total(N) | Univariate analysis | |  | Multivariate analysis | |
| --- | --- | --- | --- | --- | --- | --- |
|  |  | Hazard ratio (95% CI) | P value |  | Hazard ratio (95% CI) | P value |
| **Gender** | 179 |  |  |  |  |  |
| Female | 80 |  |  |  |  |  |
| Male | 99 | 0.813 (0.541 - 1.222) | 0.319 |  |  |  |
| **Age** | 179 |  |  |  |  |  |
| <= 65 | 94 |  |  |  |  |  |
| > 65 | 85 | 1.285 (0.853 - 1.937) | 0.230 |  |  |  |
| **Race** | 175 |  |  |  |  |  |
| White | 158 |  |  |  |  |  |
| Asian&Black or African American | 17 | 0.857 (0.430 - 1.711) | 0.663 |  |  |  |
| **Pathologic T stage** | 177 |  |  |  |  |  |
| T1&T2 | 31 |  |  |  |  |  |
| T3&T4 | 146 | 2.035 (1.079 - 3.838) | 0.028 |  | 2.132 (0.780 - 5.825) | 0.140 |
| **Pathologic N stage** | 174 |  |  |  |  |  |
| N0 | 50 |  |  |  |  |  |
| N1 | 124 | 2.161 (1.287 - 3.627) | 0.004 |  | 2.581 (1.358 - 4.905) | **0.004** |
| **Pathologic M stage** | 85 |  |  |  |  |  |
| M0 | 80 |  |  |  |  |  |
| M1 | 5 | 0.773 (0.185 - 3.227) | 0.724 |  |  |  |
| **Pathologic stage** | 176 |  |  |  |  |  |
| Stage I | 21 |  |  |  |  |  |
| Stage II&Stage III&Stage IV | 155 | 2.309 (1.059 - 5.033) | 0.035 |  | 0.332 (0.078 - 1.406) | 0.134 |
| **Histologic grade** | 177 |  |  |  |  |  |
| G1 | 31 |  |  |  |  |  |
| G3&G4&G2 | 146 | 2.174 (1.145 - 4.126) | 0.018 |  | 1.824 (0.950 - 3.502) | 0.071 |
| **PYGB** | 179 |  |  |  |  |  |
| Low | 89 |  |  |  |  |  |
| High | 90 | 1.964 (1.287 - 2.997) | 0.002 |  | 1.743 (1.125 - 2.699) | **0.013** |
